# Proteins and proteases of Prader–Willi syndrome: a comprehensive review and perspectives

**DOI:** 10.1042/BSR20220610

**Published:** 2022-06-17

**Authors:** Sanjukta Basak, Ajoy Basak

**Affiliations:** 1 Department of Pediatric Endocrinology, The Hospital for Sick Children, University of Toronto, Toronto, Canada; 2 Department of Pathology and Laboratory Medicine, Faculty of Medicine, University of Ottawa, Ottawa, Canada; 3 Department of Chronic Diseases, Ottawa Hospital Research Institute, The Ottawa Hospital, University of Ottawa, Ottawa, Canada

**Keywords:** Chromosome 15 defect, Hyperphagia, Hypotonia, Prader Willi Syndrome, Proteases, Proteins

## Abstract

**Prader–Willi Syndrome (PWS)** is a rare complex genetic disease that is associated with pathological disorders that include endocrine disruption, developmental, neurological, and physical problems as well as intellectual, and behavioral dysfunction. In early stage, PWS is characterized by respiratory distress, hypotonia, and poor sucking ability, causing feeding concern and poor weight gain. Additional features of the disease evolve over time. These include hyperphagia, obesity, developmental, cognitive delay, skin picking, high pain threshold, short stature, growth hormone deficiency, hypogonadism, strabismus, scoliosis, joint laxity, or hip dysplasia. The disease is associated with a shortened life expectancy. There is no cure for PWS, although interventions are available for symptoms management. PWS is caused by genetic defects in chromosome 15q11.2-q13, and categorized into three groups, namely ***Paternal deletion*, *Maternal uniparental disomy***, and ***Imprinting defect*.** PWS is confirmed through genetic testing and DNA-methylation analysis. Studies revealed that at least two key proteins namely ***MAGEL-2*** and ***NECDIN*** along with two proteases ***PCSK1*** and ***PCSK2*** are linked to PWS. Herein, we summarize our current understanding and knowledge about the role of these proteins and enzymes in various biological processes associated with PWS. The review also describes how loss and/or impairment of functional activity of these macromolecules can lead to hormonal disbalance by promoting degradation of secretory granules and via inhibition of proteolytic maturation of precursor-proteins. The present review will draw attention of researchers, scientists, and academicians engaged in PWS study and will help to identify potential targets and molecular pathways for PWS intervention and treatment.

## Introduction

During the past several decades, a large number of review articles on Prader–Willi Syndrome (PWS) have been published in the literature describing various aspects of the disease [1–10]. Despite these publications that provided useful information and knowledge about the disease, there exist only a limited number that are focused specifically on key proteins and enzymes that are linked to various pathophysiologies of the disease [11–13]. The present review summerizes the latest research findings about the characterizations and implications of these proteins and enzymes in molecular and biochemical pathways of PWS leading to a range of pathological consequences. This summary will help to enhance our knowledge about the cause of pathophysiologies associated with PWS and assist in developing an effective therapeutic strategy for intervention of the disease. For a comprehensive evaluation, understanding and perspectives of the disease which is reflected from neonatal (sometimes even prenatal) period of the baby to adulthood and beyond [14–16]. With increasing age and growth of the baby, many complications and abnormalities develop that may include growth retardation, neurological impairment, behavior abnormality, as well as anomalies in physical stature and appearance. Extensive research in sixties and seventies revealed many details of these and additional health abnormalities with PWS patients (reviewed in [17]). Later on, additional studies were conducted that have enriched our understanding about the disease [1,2].

## History

PWS was first officially described in the literature by three Swiss endocrinology doctors, **Andrea Prader, Alexis Labhart,** and **Heinrich Willi** in 1956 based on abnormal symptoms and clinical features of nine children patients who were under their care [18]. They are the first to observe the features, abnormalities, and health disorders that became the vital part of what is now known as PWS in medical world. It may be pointed out that nearly 70 years earlier back in 1887, **Dr. Langdon-Down** noticed in a girl patient several abnormal features such as mental impairment, short physical structure, hypogonadism, excessive feeding pattern, and high degree of obesity, which he termed as ‘**Polysarcia**’ [11]. Following their first report, Prader and his colleagues later described a series of patients with similar phenotypes. In the literature, it was also termed as **HHHO (Hypogonadism, Hypotonia, Hypomentia**, and **Obesity)** because of the presence of these hallmark features in these patients [19–21].

## Global statistics

PWS occurs in one out of every 12000–15000 live births. There is considerable geographic variation. For example in the U.K., the incidence is the lowest as one in 52000 [22]. In the U.S.A., the average incidence ranges from one in 16000 to 25000 [23] depending on the region. In rural Sweden, the rate is very high with prevalence of one in 8000 [24]. On the contrary, it is one in 16000 in Japan [25]—almost the same as global average figure [26,27]. PWS affects both genders equally in terms of degree of severity, aggressiveness, display of symptoms, and fatality. At present, globally the total number of people affected by this disease per year varies roughly from 350000 to 400000 individuals.

## Features and symptoms

The symptoms and severity of PWS can vary significantly from one individual to another. Many features of the disorder may develop slowly over time or can be subtle. It is important to note that affected individuals may not have all of the symptoms discussed below. PWS is typically identified in newborn period and is characterized by small for gestational, poor sucking, and hypotonia. Male infants with PWS may also show hypogonadism, genital hypoplasia, and undescended testes. As children grow, they develop symptoms of hyperphagia resulting in easy weight gain. The onset of hyperphagia most commonly begins between ages 2 and 8 years, but are variable in terms of onset and intensity. Individuals with PWS lack normal hunger and satiety cues, and food-seeking behaviors are very common. In addition, the metabolic rate of persons with PWS is lower than normal. Left untreated, this combination of problems leads to morbid obesity and its many complications. Children with PWS often exhibit global developmental delay, typically sitting by age 12 months and walking by 27–35 months. They may also experience delayed speech and speech apraxia. All PWS individuals exhibit some grade of cognitive challenges with measured IQs (Intelligence Quotients) ranging from low normal to moderate intellectual disability. They may also exhibit learning disabilities. In addition, they experience a variety of behavioral issues that include, skin picking, emotional dysregulation, aggressiveness, rigid attitudes, as well as manipulative and obsessive–compulsive behaviors. Adults with PWS are at increased risk for mental illness. Endocrine manifestations include growth hormone deficiency/short stature, hypothyroidism, adrenal insufficiency, and delayed puberty. From a respiratory perspective, sleep disturbances with excessive daytime sleepiness can occur. With the development of obesity, obstructive sleep apnea may occur. From an orthopedic perspective, due to joint laxity and hypotonia, ankle supports may be needed. They can also develop hip dysplasia and scoliosis. Due to dental crowding and decreased salivary flow, they are at higher risk of cavities, enamel hypoplasia, periodontal disease, delayed tooth eruption, candidiasis, and oral lesions. Data from the Global PWS Registry show that the most common vision issues in PWS are nearsightedness, and strabismus [27–31].

## Causes and classification

### Genetic

Accumulated research, various clinical and medical studies revealed that PWS is caused by genetic aberration involving chromosome 15. **Ledbetter** and his group in 1981 first detected microdeletions within this chromosome at a specific site for PWS patients [5,32–34].

### Classification of PWS

PWS has been classified into three separate categories based on abnormal genetic type.

**Figure 1:**
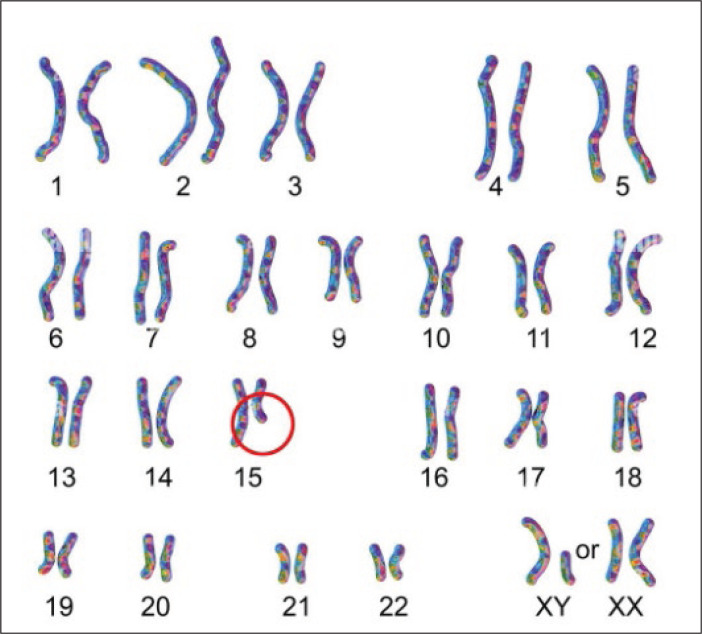
The karyotype image showing chromosomal abnormality in PWS disease Note the red circle indicating that the defect lies with long-arm bands of chromosome 15. (Taken from: https://www.sciencephoto.com/media/1127811/view/karyotype-of-prader-willi-syndrome-illustration).

**Figure 2:**
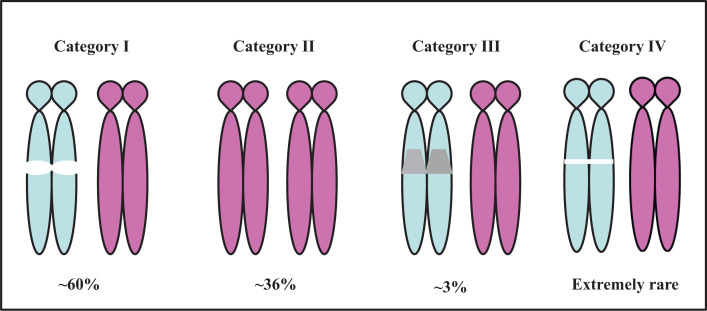
Genetic classes of PWS and their average frequencies Category I: PD; Category II: MUPD; Category III: ID. A fourth type (Category IV) caused by deletion of gene SNORD 116 (Type IV) is extremely rare. The figure represents chromosome 15. P indicates the paternally inherited chromosome 15, and M indicates the maternally contributed one. Type III occurs when there is biparental inheritance, but the paternally inherited chromosome 15 is imprinted in the manner typical of the maternal chromosome 15 (i.e., relevant genes are not expressed).

- **Category-I**: **Paternal Deletion (PD)**: 65–70% of PWS patients have a deletion or missing of a segment in paternal chromosome 15 within the proximal long arm between q11.2–q13. (Figure 1). In normal circumstance, each parent transfers one copy of chromosome 15 to their offspring but in case of most PWS children, the father’s chromosome 15 q11.2-q13 segment is not transferred. In this genetic defect, there is complete interstitial deletion of q11.2-13 segment of father’s chromosome 15 (break points q11.2 and q13) in the offspring while the copy from mother is transferred normally and is fully turned on (active). This segment encompasses the region between break point (BP) hot spots BP1 and BP3 (about 6 Mb size). This is known as deletion of type-I. Alternatively in rare cases, there is a deletion of 5.3 Mb size encompassing the region between BP1 and BP2. In extremely rare cases, there is a deletion of only 118 Kb segment encoding the protein SNORD116. Some consider it as category IV of genetic defect of PWS, others place it in ‘PD’ category. It is also noted that father’s chromosome 15 is transferred with the segment q11.2-13 fully silenced (or turned off and hence inactive). In all cases, both parents are normal and the deletion occurred as a *de novo* event. Some studies suggest increased behavioral difficulties for Type 1 (completely deleted) compared with Type 2 deletion (silenced) patients [35,36].
- **Category-II: Maternal Uni-Parental Disomy (MUPD):** In 25–30% cases of PWS, the individual inherits both copies of chromosome 15 from maternal source, instead of having one copy from father and one copy from mother. The chromosomal content is not altered in uniparental disomy and therefore disease may develop if the chromosome linked to the disomy contains ‘imprinted genes’, whose expression is highly gender-specific. Usually, both genes are ‘turned on’ meaning functionally active. However, some genes may be preferentially ‘turned off’ meaning silenced depending on which parent contributed the gene to the child (genetic imprinting). Genetic imprinting is controlled by chemical switches via DNA-methylation and other chemical changes at the DNA level. Correct genetic imprinting is crucial for normal growth and development of the baby. Defective imprinting is one of causes for PWS disorders. In general, older mothers are more probable to cause UPD than younger mothers and this could be related to the fact that older eggs are likely to have more errors in the chromosome [37]. At present, the exact reason for alteration of chromosome 15 is not known. It is also not understood whether the defect happens during egg or sperm production, maturation, or during the conception [38]. It may be pointed out that some kind of relation exists between the nature of genetic defect and the abnormalities. PWS patients having UPD exhibit a slightly improved mean intelligence quotient compared with those having deletion. They display milder behavioral abnormalities but are likely to display an enhanced degree of autistic features and mental retardation compared with deletion group. In general, these babies are born late.
- **Category-III: Imprinting Defect (ID)**: The remaining approximately 1–3% of PWS cases are due to defect in DNA-imprinting center (Figure 2). PWS individuals having defect in DNA-imprinting are more comparable with UPD group in terms of abnormalities [39,40].
- In addition to above, genetic abnormalities of PWS, a small group shows a very rare type of deletion caused by ‘**Chromosomal Translocation**’. In this case, a segment of one chromosome breaks and becomes attached to another causing a rearrangement. In spite of all defective genetic scenarios as mentioned above, it is possible that within each defective genetic group, there can be significant phenotypic variation. Knowing the exact genetic defect of PWS individuals is not helpful in diagnosing the actual symptoms that may show up for any individual [41–46].

## Molecular mechanism and pathophysiology

In order to better understand the molecular mechanism and pathophysiology of PWS, studies have been conducted to identify proteins that are encoded by the missing part of chromosome 15 and associated proteases. This part of the gene represents about 6 Mb (Mega bases, 1 Mb = 1000000 base pair) that comprises six small nucleolar RNA genes (which are noncoding) and six protein-coding genes. The associated coded proteins have been identified and these are: **(i) Melanoma Antigen L-2 (MAGEL-2), (ii) NECDIN (NDN), (iii) Makorin Ring Finger Protein-3 (MKRN-3), (iv) Nuclear Pore Associated Protein-1 (NPAP-1), (v) Small Nuclear Ring Finger (SNURF), and (vi) Small Nuclear Ribonucleoprotein N (SNRPN)** [47–50]. Among these proteins, MAGEL-2 and NECDIN have been strongly implicated in some of the pathophysiologies and abnormalities observed with PWS individuals. The rest of the proteins that also include **IPW** (ImPrinted gene in the Prader–Willi syndrome region) [51], **PWRN1** (Prader–Willi Region Non-protein coding RNA-1) [52], **SNORD-116** (Small NucleOlar RNA, C/D box 116) (53) have been either proposed or inadequately linked to some conditions like neurological abnormalities of PWS based on limited clinical and/or model animal studies. In particular, microdeletion in SNORD-116 may be linked to PWS conditions of some individuals but not for others [53].

## Proteins and proteases of PWS

### Proteins

#### MAGEL-2:

**Figure 3:**
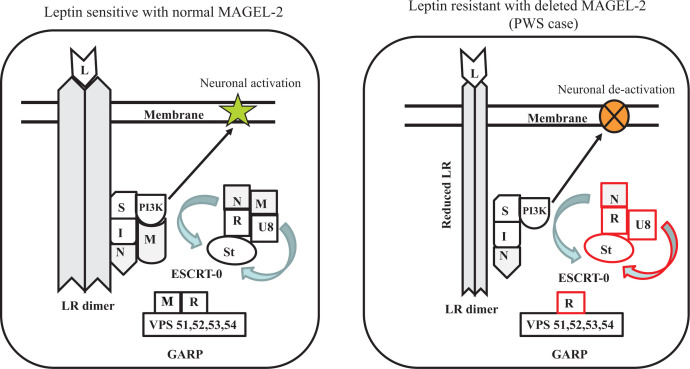
Schematic diagram showing the regulation of leptin receptor activity mediated by **MAGEL-2** The panel on the **left** shows how MAGEL-2 participates in the biological pathway to keep appetite under balance. The panel on the **right** indicates the effect when MAGEL-2 is absent or silenced. Loss of MAGEL-2 causes change in ubiquitination of proteins in leptin receptor internalization pathway, with affected proteins outlined in red (**right**). Proteins are labeled as follows: leptin (**L**), leptin receptor (**LR**), SH2B1 (**S**), IRS4 **(I)**, NECDIN **(N)**, MAGEL-2 **(M)**, phosphatidylinositol-3 kinase **(PI3K),** RNF41 **(R),** USP8 **(U8),** Stam1 **(St).** MAGEL-2 and NECDIN in combination regulate LR sorting and degradation through a dynamic ubiquitin-dependent pathway. Loss of either of these proteins may uncouple LR from ubiquitination pathways, providing a mechanism for obesity in PWS.

**Figure 4:**
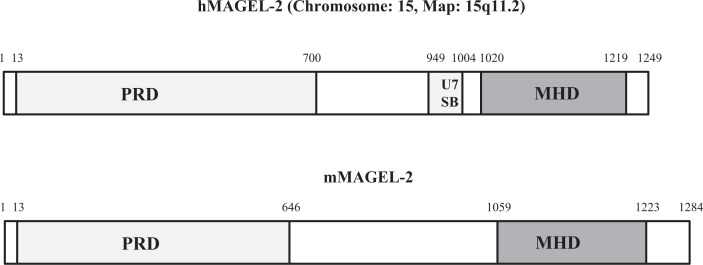
Schematic structure of human (h)MAGEL-2 (ACCESSION NO: NP_061939) and mouse (m)MAGEL-2 (ACCESSION NO: NP_038807, XP_622091) **Upper Panel**: Human MAGEL-2 contains a proline-rich domain (**PRD**: residues 13–700), USP7-binding segment (**U7BS**: residues 949–1004), and MAGE homology domain (**MHD**: residues 1020–1219). A number of truncating variants of hMAGEL-2 have been reported. These (frameshift and nonsense variants) are mostly located in PRD, followed by **MHD** and U7BS domains. The mutation hotspot is located at nucleotides residue 666aa. **Lower Panel**: Mouse **MAGEL-2** contains proline-rich region (residues 13–646) and **MHD** (residues 1059–1223) but no U7BS domain.

**MAGEL-2** along with **NECDIN** are key cellular proteins that have been strongly linked to appetite disbalance observed with PWS patients. Studies have indicated that lack or absence of these proteins is the primary cause for uncontrolled appetite and excessive overeating behavior of PWS individuals. it has been established by a number of *in vitro, in vivo* and animal knock out studies that these proteins enhance the level of leptin receptor on the cell surface via ubiquitination pathways [54,55]. MAGEL-2 null mice are obese and do not respond to leptin in hypothalamic pro-opiomelanocortin neurons, suggesting dysregulation of leptin receptor activity. Research also showed that MAGEL-2 knockout mice display a much reduced level of leptin receptor in their hypothalamus and altered expressions of several key proteins like **RNF-41** (E3 ubiquitin ligase RING finger protein-41), **USP-8** (Ubiquitin-specific protease 8), and **STAM-1** (Signal-Transducing Adapter Molecule), which are associated with ubiquitination pathway that controls the activity of leptin receptor. MAGEL-2 helps to promote the level of cell surface leptin receptor by protecting its degradation. Leptin receptor binds with **MAGEL-2** via **NECDIN** and forms a multiprotein complex with **RNF-41** and **USP-8.** It is also noted that several loss of function mutant variants of MAGEL-2, which suppress its binding with **RNF-41**, decreases leptin receptor level leading to increased appetite. The above biochemical pathway is depicted in a simple visual schematic manner in Figure 3. The figure shows how loss of MAGEL-2 function or its absence may cause alterations in the abundance and ubiquitination of proteins involved in internalization pathway of leptin receptor. The proteins affected are shown in red color (right panel of Figure 3). Human (h) MAGEL-2 belongs to MAGE (Melanoma Antigen Gene) family proteins that are known to control functional activity of ubiquitin ligase. This family of proteins are also described as tumor-associated antigens and consist of >60 genes, which share a conserved MAGE homology domain. They play role in normal development and tumor progression, being found to be highly expressed in cancer tissues [56]. This is linked to dysregulation of ubiquitin pathway as mentioned above. hMAGE-2 is a 1249 amino acid (aa) long protein that contains a number of characteristic domains as described in Figure 4. The protein contains a **‘Proline-Rich Domain’** (PRD 13aa-700aa), **‘USP7 Binding Segment**’ (U7BS 949aa-1004aa) (deubiquitinating enzyme), and the crucial **‘MAGE Homology Domain**’ (MHD: 1020aa-1219aa) [57]. The most critical domain of interest for MAGEL-2 is U7BS via, which it interacts with **USP7**. A number of truncating variants of MAGEL-2 due to frameshift and nonsense mutations have been reported that are likely responsible for various neurological and related anomalies found in **SYS** (**Schaf–Yang Syndrome**) [57], PWS and other related diseases. These truncations occur around amino acid position approximately 666 as shown in the figure. Most observed mutations have been reported within PRD domain near its C-terminal region (653aa-708aa). The next most hotspot mutation sites are located in MHD followed by U7SB domains (Figure 4). It is interesting to note that mouse MAGEL-2 does not contain any U7SB domain—unlike in human and its impact is not clear.

**Figure 5:**
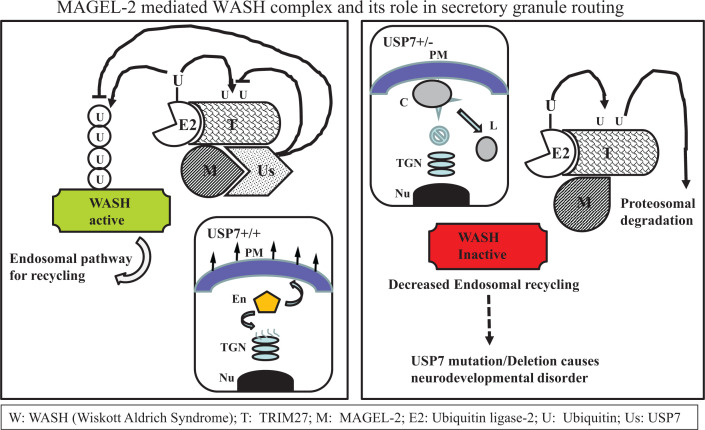
Biochemical pathway showing the role of MAGEL-2 mediated WASH complex in SG (Secretory granuke) routing and potential implication **Left**: Schematic diagram showing the pathways of protein sorting to SG via endosomal pathway and WASH complex in presence of MAGEL-2 protein. **Right**: Shows the pathway of WASH complex via degradative lysosomal pathway when MAGEL-2 is absent or silenced. The abbreviations of various proteins in WASH complex are indicated on the right side of the figure. Abbreviations: C: cargo; En: endosome; L: lysosome; Nu: nucleus; PM: plasma Membrane; TGN: trans Golgi network.

It is observed that inactivation (part or complete) of MAGEL-2 or lack of it occurs in people with **Prader–Willi Syndrome, Schaaf-Yang syndrome, Arthrogryposis**, or **Opitz-C Syndrome,** all of which display some common symptoms such as excessive feeding pattern and massive obesity. Therefore, they are likely to have disruption in their molecular pathway associated with hunger and appetite. This observation is highly significant for PWS where MAGEL-2 is missing in most individuals (particularly the PD or Parental Deletion type). It is now well established that MAGEL-2 not only functions in cancer but also mediates in endosomal trafficking of 500 kDa protein called ‘**WASH complex**’ that includes a variety of proteins such as **WASH1 (Wiskott Aldrich Syndrome 1), FAM21** (which contains 21 copies of a novel amino acid motif: ‘L-F-[D/E]_3-10_-L-F’), **Strumpellin**, **SWIP (Strumpellin and WASH-Interacting Protein),** and **CCDC53 (Coiled Coil Domain Containing p53).** This complex prevents lysosomal degradation of **SG** (Secretory Granule) and dense SG (type of organelles) proteins [57]. The formation of this multiprotein complex is responsible for routing the protein to endosomal pathway. Lack of any of these proteins, especially MAGEL-2 will promote destruction key proteins via lysosomal pathway. In PWS, the loss of MAGEL-2 leads to reduced production of neuropeptide such as **Oxytocin (Oxt)**, **Arginine-vasopressin (Avp),** and **Somatostatin (Sst)** (stored in SG)—which is the cause of hormonal related neurological and intellectual disbalance. Degradation of other hormones such as **growth hormone**, **luteinizing hormone**, **thyrotropin-releasing hormone**, **proenkephalin**, **chromogranins**, and **prolactin**, which rely on neurosecretory input from the hypothalamus also, occurs leading many neurological and other distress seen in PWS patients. This conclusion is consistent with the observation that SG and neuropeptide abundance are significantly altered in MAGEL-2 knockout mice [55]. It is thus concluded that MAGEL-2 plays a significant major role in hypothalamic neuroendocrine function and cellular disturbance. Its deficiency disrupts this event and thereby contributes to PWS pathophysiology [58]. Two key pathways involving MAGEL-2 have been described. One involves SG containing hormone pathway and the other leptin-associated hunger pathway as shown in the figure, causing hormonal disbalance and excessive appetite pattern as observed with PWS patients (Figures 3 and 5). It is now evident that the normal function of MAGEL-2 is to keep the general maintenance and activity in the cell under control. Overall, it acts as an important part of a large protein complex recycling of vesicles in the cell in right direction. This allows receptors, signaling molecules, hormonal and neuronal peptides, as well as other proteins to be stored in vesicles and trafficked to the right places. This is disrupted in PWS due to silencing or deletion of MAGEL-2 [59].

#### NECDIN

**Figure 6:**
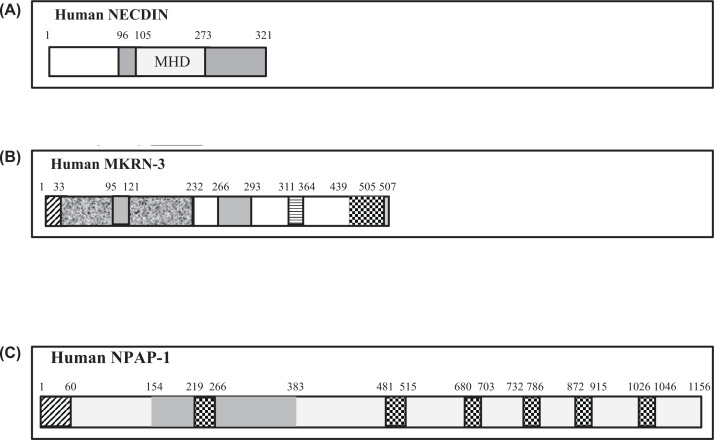
Amino sequences of human NECDIN, human MKRN-3 and human NPAP-1 in schematic forms showing their various characteristic domains (**A**) Schematic diagram showing the structure of human NECDIN protein (ACCESSION number: NP_002478) with its various characteristic domains. Disordered: 1–96; MAGEL homology domain (**MHD**): 105–273. (**B**) Schematic diagram showing the structure of human MKRN-3 (Makorin Ring Finger Protein-3) (ACCESSION number: AAH44639); Disordered: 1–33; DNA polymerase III subunit γ and τ domain III: 34–232: Zinc-finger domain: 95–121: Makorin-type Cys–His domain: 266-–293: Zinc-binding segment: 311-364, (Sites: 311, 314, 335, 337, 340, 343, 361, 364); E3 ubiquitin-protein ligase makorin-1: 439–505. (**C**) Schematic diagram showing the structure of human NPAP-1 protein (ACCESSION number: NP_061831) with various characteristic domains. Regions of interest: 1-–60; 219–266; 481-–515; 680–703; 872–915; 1026-–1046. POM-121 family domain: 154–383.

The second most important protein associated with PWS is NECDIN (NDN). This is a DNA-binding protein that helps in the maturation and activation of neurons following termination of cell division and elevation of axonal outgrowth [60]. This is facilitated via its interaction with a number of key partner proteins that include **NGF** (Nerve Growth Factor), **MAGEH-1** (Melanoma Associated Antigen Family H-1) and **MAGEL-2.** This helps to protect from degradation the protein **FEZ-1** (Fasciculation and Elongation Zeta 1) protein, which stimulates axonal outgrowth [61]. NDN is likely associated with hypogonadotropic hypogonadism condition as observed in PWS patients. It is needed for the function of neurons associated with **GnRH** (gonadotropin releasing hormone). Deficiency, absence, or silencing of NDN is the primary cause of PWS etiology linked to hypogonadism observed with adolescent PWS children. In this case, sex glands (called gonads) are underdeveloped and generate little or no sex hormones. Overall speaking, it is now established that NDN plays role in intracellular processes associated with neurite outgrowth and negative regulation of axonal outgrowth, migration, and survival of Embryonic Sympathetic Neurons. Human NECDIN is a 321 aa long nuclear protein that belongs to MAGE family protein. The region encompassing the residues from 105aa to 273aa has been identified as the MAGE Homology Domain (MHD)—a domain characteristic of all MAGE proteins (Figure 6A) [62]. Its difference in structure from corresponding mouse NECDIN, which is lacking U7SB domain is noticeable in the figure.

#### MKRN-3

**MKRN-3** is a zinc finger protein that is specifically linked to puberty regulation in adolescent males and females. It likely plays role in sex hormones (androgens, estrogens, and progesterone) expression and regulation. Lack of this protein may cause improper genital development and/or late puberty for PWS adolescent individuals. However, the exact mechanism and biological pathway remain unclear. It is interesting to note that frameshift mutations leading to its truncation along with missense mutations have been found in individuals who exhibit central precocious puberty similar to that noted with PWS patients [63]. The involvement of MKRN3 gene and associated protein has been proposed based on observed phenotypes upon its loss of expression or its complete deletion. Thus, it is evident that missing or silencing of MAGEL-2 and NDN alone is not sufficient to explain all the phenotypes of PWS individuals [64]. Human MKRN-3 is a 507 aa long protein that has multiple domains including the most important Zn^+2^-binding sites, which comprise residues from 311aa to 364aa (see Figure 6B). So far studies revealed that absence or silencing of this protein is the primary cause for defects and anomalies in reproductive function and organ development seen with adolescent PWS individuals [65].

#### NPAP-1

**NPAP-I or nuclear pore-associated protein-1** is a paternally expressed imprinted gene located in the region between q11–q13 of chromosome-15. Since PWS originates from the deletion or functional loss of same segment of human genome, it is suggested that NPAP-1 protein may be implicated in some anomalies found in PWS individuals. The mechanism and the details of imprinting consequences of this protein is not fully understood. A thorough investigation on the evolutionary origin of this protein established that the gene is explicit to primates and are absent from chromosome-15 q11–q13-orthologous regions in all nonprimate mammals [54]. Human NPAP-1 protein is a large 1156aa long protein that contains a characteristic POM-121 family domain segment. It also contains seven domains as indicated, which are considered as segments of interest (Figure 6C). This protein belongs to a POM-121-related family of retrogenes. Retrogene is a processed copy of another gene and is usually nonfunctional, inactive, and regarded as biologically insignificant segments [66,67].

#### SNRPN and SNURF

The full-length original gene **SNRPN**, abbreviated for **S**mall **N**uclear **R**ibonucleoprotein **P**olypeptide **N**, represents two specific functional proteins encoded by the genes **SNURF** short for **SN**RPN **U**pstream **R**eading **F**rame and **SNRPN**. These two are separated by a noncoding segment of nearly 600 kb in size, termed as **SNHG-14** (**S**mall **N**uclear **H**ost **G**ene-14). The biological role of **SNURF** protein that consists of first three exons 1–3 in PWS is not fully understood. In contrast, **SNRPN** that contains exons 4–10 produces a nonessential protein called **SMN** (**S**urvival **M**otor **N**euron), which plays important role in mRNA splicing [68]. It has been suggested that **SNHG-14** induces the upstream exons of **SNRPN** gene and encompasses a range of RNA segments that display a wide variety of putative functions. In particular, two snoRNAs (small nucleolar RNAs) have been described that are generated via activation mediated by **SNHG-14.** Moreover, another noncoding RNA molecule called **SNORD-116** (**S**mall **N**ucle**o**lar **R**NA of C/**D** box type) has been described that regulates other approximately 12 snoRNAs (mostly 150 nucleotides in size). **SNORD-116** is strongly expressed in brain tissues in normal individuals but is lacking in PWS patients suggesting its strong linkage to PWS. In fact, its absence has been implicated to several key abnormalities of PWS patients such as feeding difficulties during infancy, weight gain after 2 years of age, extreme appetite, and developmental defect [69].

### Epigenetic cause

Studies have shown that **PWS** syndromes are also linked to epigenetic regulation of chromosome 15 locus 15q11-q13. Thus, understanding the mechanism of repression of this locus inherited from mother is key from point of view of therapeutic intervention of **PWS**. A significant number of PWS individuals do not have the paternal 15q11-q13 locus but inherit the same intact from mother that remains epigenetically silent. Gene expression at this locus is mostly regulated by **PWS-IC** (Prader–Willi Syndrome Imprinting Center). This is described as the master regulator of **MAGEL-2, MKRN-3**, and **NDN** proteins, which remain unmethylated in paternal allele but is likely methylated in maternal allele. This differential methylation occurred in the germline as the result of transcriptional activation of an oocyte-specific promoter(s) upstream of SNRPN. This mechanism establishes the original silencing of maternal chromosome. Recently, the existence of separate somatic imprints in this silencing activity has been uncovered [70,71]. The epigenetic DNA-methylation that is mediated by DNA methyltransferase enzyme has been considered as a therapeutic target for possible management of PWS. The first proof of principle of this approach was obtained when it was shown that a DNA methyltransferase inhibitor, ‘**5-azadeoxycytidine**’ was able to demethylate the PWS-IC leading to activation of the maternal genes. Therefore, activation of the normally silent such maternal gene inhibitory compound may offer a potential therapeutic intervention and treatment for PWS patients [70,71]. Recently, a protein named **‘ZNF-274**’ (Zinc Finger Protein 274) that contains five Zinc-finger domains has been identified [72], which plays important role in the suppression of maternal chromosome-15q11-q13 allele. It forms a silencing complex with other key proteins such as SETDB1 and is associated with enhanced expression in maternal allele. The study results support the notion that ZNF274 recruits SETDB1 to maternal SNORD116, where it deposits another protein H3K9me3 and contributes to repression of the maternal allele. ZNF-274 knock study provided additional support for this hypothesis [73,74].

### Proteases of PWS and their defects

#### PCSK1, PCSK2, and CPE

**Figure 7:**
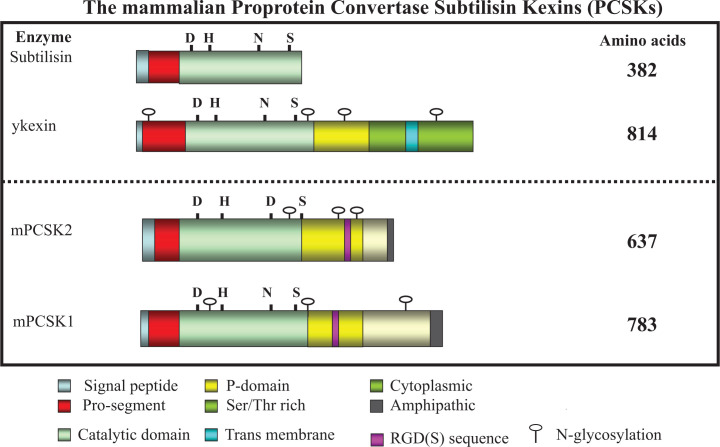
Schematic diagram showing the characteristic domains including the key catalytic, pro, and other domains of human PCSK1 and PCSK2 enzymes The domains are compared with the corresponding bacterial subtilisin and yeast (y) kexin enzymes (**shown on the top panel**). The key catalytic amino acid residues, D (Asp), H (His), S (Ser), and N (Asn) are depicted in the figure.

One key aspect of PWS is the significant loss of Secretory Granules (SG), which are large dense core vesicles and specialized intracellular organelles. SGs store many endocrine hormones and neuronal polypeptides in their active forms following their proteolytic processing. They are synthesized in ER (Endoplasmic Reticulum), travel through TGN (Trans Golgi Network) apparatus where change of pH and proteolytic activation take place. Following maturation, they are packaged and stored in SG before secretion. Following signal and stimulation, these bioactive hormones and neuropeptides are released either in regulated or in constitutive pathway. PWS individuals lack many of these key hormones and neuropeptides as SGs are gradually destroyed and this is the primary cause for many of the observed symptoms and abnormalities of PWS. It was also noted that in PWS there is not only degradation of SGs but there is also significant lack of maturation of inactive precursor prohormones, proproteins, and proneuropeptides. This is due to reduced level of any one of the three enzymes that include **Proprotein Convertase Subtilisin Kexin type 1** and **type 2 (PCSK1 and PCSK2)** (reviewed in [75,76]) as well as **Carboxy Peptidase E (CpE)** [77]. This caused decreased processing of proneuropeptides and prohormones to the corresponding mature active forms. The loss or impaired protease activity of PCSK1 (known as PC1 at that time) in particular has been reported in human and this led to high appetite causing obesity and diabetes [78]. This finding is also confirmed by studies involving PCSK1 knockout mice models (reviewed in [78,79]). Both PCSK1 and PCSK2 belong to the same PCSK enzyme family and are mostly present in endocrine tissues including brain. The characteristic structural features, the crucial catalytic domain with key amino acid residues **[Asp (D), His (H), and Ser (S)]** responsible for the protease activity along with other domains of PCSK1 and PCSK2 are shown in Figure 7. So far impaired PCSK1 activity has been strongly linked to Prader–Willi Syndrome and associated childhood obesity [80]. However, the precise cause of impairment is not clear, although later on it was linked to mutation of catalytic residue/s. The other possibility is down-regulation of PCSK1 expression due to reason not yet understood.

Consistent with this finding, previous studies indicated reduced hormone processing activity in MAGEL-2-deficient mice, including significantly low level of active orexin (A hormonal neuropeptide that regulates arousal, wakefulness, and appetite) in circulation. Hormone imbalance such as increased unprocessed precursor proteins such as pro-orexin, diminished expression of oxytocin, and decreased production of growth hormones are all associated with PWS. These are likely instigated by impaired protease activity of PCSK1 enzyme. The same reasoning may also hold true for reduction in secreted insulin level in blood for PWS deletion model mouse [64]. Similar findings were also noted in humans, where PWS patients were found to exhibit much reduced levels of hormones like vasopressin and oxytocin. Improper and defective processing of neuropeptides and their secretion are consistent with hormonal imbalance noted in PWS individuals. This contributes to various conditions of PWS such as hyperphagic obesity, hypogonadism, growth hormone deficiency, hyperghrelinemia, and hypoinsulinemia. In agreement with the above notion of reduced production of neuropeptides in PWS as well as in MAGEL-2 knockout model animals, an administration by injection of oxytocin in postnatal condition is able to rescue serious feeding behavior in MAGEL-2-knockout mice [79,80]. MAGEL-2 null mice phenotypes are in consistent with many PWS features. The lack of prohormone processing in PWS individuals is in line with that observed during experiments with MAGEL-2 null mice where distinct abnormality in SG in hypothalamic tissues has been observed. A detail proteomics analysis of knockout tissues demonstrated reduced levels of important neuropeptides such as **Oxt (Oxytocin), Avp (Arg-vasopressin),** and **Sst (Somatostatin).** Latter observation has been linked to neurological abnormality in PWS individuals [1–5]. Moreover, null pituitary tissues showed diminished levels of GH (growth hormone), LH (luteinizing hormone), and prolactin, which are implicated in neurosecretion [81]. Since hypothalamus/pituitary axis may regulate function of other tissues, therefore, associated organs may be indirectly affected by the absence of MAGEl-2. Experiments revealed improper regulation of metabolic genes in liver and white adipose tissues in MAGEL-null mice compared with control. Thus, in conclusion, it was stated that SG and neuropeptides are substantially diminished in null mice—a reminiscent of PWS in human.

**Figure 8:**
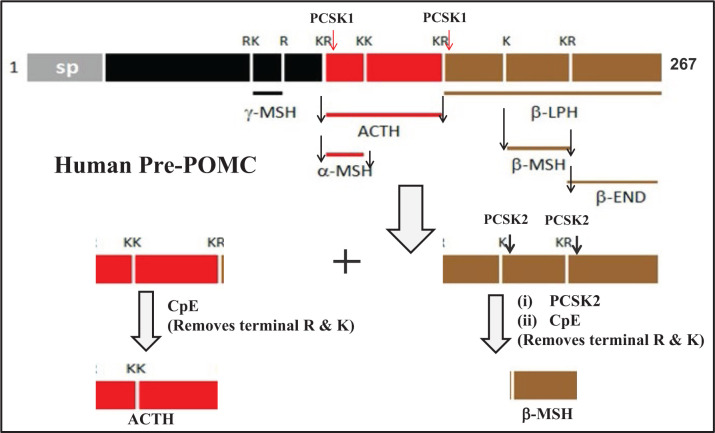
POMC maturation via the proteolytic actions of enzymes PCSK1, PCSK2 and CpE Schematic presentation of POMC (Pro-opiomelanocorticoid) cleavages by PCSK1 and /2 at single and paired basic amino acid residues (K/R), leading to the formation of various bioactive peptides like β-MSH, α-MSH, β-LPH, ACTH, γ-MSH, etc. where CpE (Carboxy peptidase E) also plays a critical role in the maturation process by cleaving off the excess basic residues at the C-terminus to generate ultimately the active hormonal peptide. (sp: signal peptide; R: arginine; K: lysine).

The role of three key enzymes **PCSK1, PCSK2**, and **CpE** in hormone production and maturation has been well documented in the literature by a large number of studies (reviewed in [81]). This is explained in a schematic manner in Figure 8, using the maturation process of a typical brain protein, **POMC** (**Pro Opio Melano Corticoid**) that generates a variety of active hormones via the conserted actions of PCSK1, PCSK2, and CpE [77]. It is therefore understandable why impaired activities of any one of these enzymes can lead to decreased levels of key hormones and neuropeptides [82]. This is one of the crucial findings for the cause of PWS disease and the associated various observed symptoms and functional abnormalities. So far deficiency of PCSK1 gene and its coded protein (enzyme) activity has been confirmed in Prader–Willi Syndrome disease and associated obesity [80,81]. *So far it is not fully known what causes reduced activities of PCSK1 and possibly PCSK2 and CpE in PWS patients. It could be plausible that some of the proteins encoded by the deleted segment of chromosome-15 may affect in a negative manner (down-regulate) the activity of the enzymes, especially PCSK1 whose deficiency has been already linked to many PWS-associated abnormalities [78,83,84].*

## Future and perspectives

The future direction of PWS research is directed toward gene therapy. Current research is more focused in finding means to activate (turn on) the **‘silent genes**’ on the maternal chromosome 15 (l). Understanding how proteins function and interact is important in this event. This is expected to open up a new horizon for more effective treatment and possibly a cure for PWS disease in the future. In the past, the treatment was directed toward supplementing the hormones, growth factors, and other bioactive peptides as nicely summarized in a recent review by Chung et al. [9].

## Concluding remark

Recent and past studies have led to enhancement of our understanding about PWS disease, its progress mechanism, various symptoms, and abnormalities. However, this disease with multifaceted symptoms that appear at various stages of its progression poses a serious challenge to researchers and scientists. The research in this field is constantly evolving and bringing in new information. Understanding the molecular pathways and identification of associated biomolecules and proteins/enzymes as well as their role are all critical part of the the disease and may be considered as possible targets for therapeutic intervention of PWS. This review summerizes the role of various key proteins and enzymes and how their lack or impaired activity can lead to defects in development, growth, appetite, metabolism, hormonal balance, endocrine, and gonad functions. Among the proteins, the functional properties of MAGEL-2 and NDN are most critical while among the proteases, PCSK1 activity is the major determinant although the impairment of other enzymes such as PCSK2 and CpE in PWS cannot be ruled out [85]. Current advancements and discoveries in gene therapy research is highly encouraging and is expected to provide hopes for better treatment of PWS individuals in coming future.

## Abbreviations

Aa: Amino acid
Avp: Arginine vasopressin
BP: Break point
CCDC53: Coiled Coil Domain Containing p53
CpE: Carboxy peptidase
GH: Growth hormone
h: Human
HHHO: Hypogonadism, Hypotonia, Hypomentia, and Obesity
ID: Imprinting defect
IPW: ImPrinted gene in the Prader–Willi syndrome region
LH: Luteinizing hormone
LR: leptin receptor
MAGE: Melanoma Antigen Gene
MAGEL-2: Melanoma Antigen L-2
MHD: MAGE homology domain
MKRN-3: Makorin Ring Finger Protein-3
MUPD: Maternal uniparental disomy
NDN: NECDIN
NPAP-1: Nuclear Pore Associated Protein-1
Oxt: Oxytocin
PCSK1/2: Proprotein Convertase Subtilisin type 1/2
PD: Paternal deletion
POMC: Pro-opiomelano corticoid
PRD: Proline-rich domain
PWRN-1: Prader–Willi Region Non-protein coding RNA-1
PWS: Prader–Willi Syndrome
RNF-41: E3 ubiquitin ligase RING finger protein-41
SG: Secretory granule
SNORD-116: Small NucleOlar RNA, C/D box 116
snoRNA: Small nuclear RNA
SNRPN: Small Nuclear Ribonucleoprotein N
SNURF: Small Nuclear Ring Finger
Sst: Somatostatin
STAM-1: Signal-Transducing Adapter Molecule
SWIP: Strumpellin and WASH-Interacting Protein
SYS: Schaf-Yang Syndrome
USP-8: Ubiquitin-specific protease 8
ZNF-274: Zinc Finger Protein 274

## References

[1] Burman P., Ritzén E.M. and Lindgren A.C. (2001) Endocrine dysfunction in Prader-Willi syndrome: a review with special reference to GH. Endocr. Rev. 22, 787–799 10.1210/edrv.22.6.0447 11739333

[2] Butler M.G., Lee P.D.K. and Whitman B. (2006) Management of Prader-Willi Syndrome, 3rd edn, Springer, New York

[3] Cassidy S., Schwartz S., Miller J.L. and Driscoll D.J. (2012) Prader-Willi syndrome. Genet. Med. 14, 10–26 10.1038/gim.0b013e31822bead0 22237428

[4] Cassidy S.B. and Driscoll D.J. (2009) Prader-Willi syndrome. Eur. J. Hum. Genet. 17, 3–13 10.1038/ejhg.2008.165 18781185 PMC2985966

[5] Griggs J.L., Sinnayah P. and Mathai M.L. (2015) Prader-Willi syndrome: from genetics to behaviour, with special focus on appetite treatments. Neurosci. Biobehav. Rev. 59, 155–172 10.1016/j.neubiorev.2015.10.003 26475993

[6] Angulo M.A., Butler M.G. and Cataletto M.E. (2015) Prader-Willi syndrome: a review of clinical, genetic, and endocrine findings. J. Endocrinol. Invest. 38, 1249–1263 10.1007/s40618-015-0312-9 26062517 PMC4630255

[7] Heksch R., Kamboj M., Anglin K. and Obrynba K. (2017) Review of Prader-Willi syndrome: the endocrine approach. Transl. Pediatr. 6, 274–285 10.21037/tp.2017.09.04 29184809 PMC5682385

[8] Butler M.G., Miller J.L. and Forster J.L. (2019) Prader-Willi syndrome - clinical genetics, diagnosis and treatment approaches: an update. Curr. Pediatr. Rev. 15, 207–244 10.2174/1573396315666190716120925 31333129 PMC7040524

[9] Chung M.S., Langouët M., Chamberlain S.J. and Carmichael G.G. (2020) Prader-Willi syndrome: reflections on seminal studies and future therapies. Open Biol. 10, 200195, pp 1-17 10.1098/rsob.200195 32961075 PMC7536080

[10] Noordam C., Höybye C. and Eiholzer U. (2021) Prader-Willi syndrome and hypogonadism: a review article. Int. J. Mol. Sci. 22, 2705, pp 1-12 10.3390/ijms22052705 33800122 PMC7962179

[11] Tennese A.A., Gee C.B. and Wevrick R. (2008) Loss of the Prader-Willi syndrome protein necdin causes defective migration, axonal outgrowth, and survival of embryonic sympathetic neurons. Dev. Dyn. 237, 1935–1943 10.1002/dvdy.21615 18570257

[12] Mian-Ling Z., Yun-Qi C. and Chao-Chun Z. (2020) Prader-Willi syndrome: molecular mechanism and epigenetic therapy. Curr. Gene Ther. 20, 36–43 10.2174/1566523220666200424085336 32329685

[13] Liu X., Luo M., Liu Q. and Yang G.C. (2021) A novel mutation in the myosin binding protein C gene in a Prader-Willi syndrome pedigree. Reprod. Sci. 28, 2718–2722 10.1007/s43032-021-00620-4 34076875

[14] Salehi P., Stafford H.J., Glass R.P., Leavitt A., Beck A.E. and Mcfee A. (2017) Silent aspiration in infants with Prader-Willi syndrome identified by videofluoroscopic swallow study. Medicine (Baltimore). 96, e9256, pp. 1–6 10.1097/MD.0000000000009256 29390364 PMC5815776

[15] Miller J.L., Lynn C.H., Driscoll D.C., Goldstone A.P., Gold J.-A., Kimonis V. et al. (2011) Nutritional phases in Prader-Willi syndrome. Am. J. Med. Genet. Part A 9999, 1–10 10.1002/ajmg.a.33951 PMC3285445 21465655

[16] McAllister C., Whittington J. and Holland A. (2011) Development of the eating behaviour in Prader-Willi Syndrome: advances in our understanding. Int. J. Obes. 35, 188–197 10.1038/ijo.2010.139 20680019

[17] Driscoll D.J., Miller J.L., Schwartz S. and Cassidy S.B. (1998) Prader-Willi Syndrome. 1998 Oct 6 [updated 2017 Dec 14]. In GeneReviews®(Adam M.P., Ardinger H.H., Pagon R.A., Wallace S.E., Bean L.J.H. and Gripp K.W., eds), pp. 1993–2022, University of Washington, Seattle, Seattle (WA), https://www.ncbi.nlm.nih.gov/sites/books/NBK1330/20301505

[18] Prader A., Labhart A. and Willi H. (1956) Ein Syndrom von Adipositas, Kleinwuchs, Kryptorchismus und Oligophrenie nach Myatonieartigem Zustand im Neugeborenenalter. Schweiz. Med. Wschr. 86, 1260–1261

[19] Van Robays J. (2016) John Langdon Down (1828–1896). Facts, Views Vis Obgyn 8, 131–136, PMCID: PMC5130304 27909572 PMC5130304

[20] Ward C. (1997) Down's 1864 case of Prader-Willi syndrome: a follow-up report. J. R. Soc. Med. 90, 694–696 10.1177/014107689709001221 9496304 PMC1296748

[21] Bohonowych J., Miller J., McCandless S.E. and Strong T.V. (2019) The global Prader-Willi syndrome registry: development, launch, and early demographics. Genes 10, 713, pp. 1–14 10.3390/genes10090713 31540108 PMC6770999

[22] Whittington J.E., Holland A.J., Webb T., Butler J., Clarke D. and Boerd H. (2001) Population prevalence and estimated birth incidence and mortality rate for people with Prader-Willi syndrome in one UK Health Region. J. Med Gen. 38, 792–798 10.1136/jmg.38.11.792 PMC1734766 11732491

[23] McCandless S.E., Suh M., Yin D., Yeh M., Czado S., Aghsaei S. et al. (2020) J. Endocrine Soc. 4, Poster604

[24] Akefeldt A., Gillberg C. and Larsson C. (1991) Prader-Willi syndrome in a Swedish rural county: epidemiological aspects. Dev. Med. Child Neurol. 33, 715–721 10.1111/j.1469-8749.1991.tb14950.x 1916026

[25] https://www.uptodate.com/contents/epidemiology-and-genetics-of-prader-willi-syndrome

[26] https://rarediseases.org/rare-diseases/prader-willi-syndrome/#:∼:text=PWS%20affects%20males%20and%20females,about%20350%2C000%2D400%2C000%20individuals%20worldwide

[27] Cassidy S.B. (2003) Prader-Willi Syndrome. NORD Guide to Rare Disorders, pp. 237–238, Lippincott Williams & Wilkins, Philadelphia, PA, https://rarediseases.org/rare-diseases/prader-willi-syndrome/

[28] Butler M.G., Kimonis V., Dykens E., Gold J.A., Miller J., Tamura R. et al. (2018) Prader-Willi syndrome and early-onset morbid obesity NIH rare disease consortium: a review of natural history study. Am. J. Med. Genet. A. 176, 368–375 10.1002/ajmg.a.38582 29271568 PMC6065257

[29] Butler M.G., Manzardo A., Heinemann J., Loker C. and Loker J. (2017) Causes of death in Prader-Willi syndrome: Prader-Willi Syndrome Association (USA) 40-year mortality survey. Genet. Med. 19, 635–642 10.1038/gim.2016.178 27854358 PMC5435554

[30] Godler D.E. and Butler M.G. (2021) Special issue: genetics of Prader-Willi syndrome. Genes (Basel) 12, 1429–1435 10.3390/genes12091429 34573411 PMC8471269

[31] Cheon C.K. (2016) Genetics of Prader-Willi syndrome and Prader-Will-Like syndrome. Ann. Pediatr. Endocrinol. Metab. 21, 126–135 10.6065/apem.2016.21.3.126 27777904 PMC5073158

[32] Höybye C. (2013) Prader-Willi Syndrome. Congenital Disorders: Laboratory and Clinical Research, 1st edn, Nova Science Publishers Inc, New York

[33] Butler M.G. (2011) Prader-Willi syndrome: obesity due to genomic imprinting. Curr. Genomics 12, 204–215 10.2174/138920211795677877 22043168 PMC3137005

[34] Rocha C. and Paiva C. (2014) Prader-Willi-like phenotypes: a systematic review of their chromosomal abnormalities. Genet. Mol. Res. 13, 2290–2298 10.4238/2014.March.31.9 24737477

[35] Butler M.G., Bittel D.C., Kibiryeva N., Talebizadeh Z. and Thompson T. (2004) Behavioral differences among subjects with Prader-Willi syndrome and type I or type II deletion and maternal disomy. Pediatrics 113, 565–573 10.1542/peds.113.3.565 14993551 PMC6743499

[36] Ledbetter D.H., Riccardi V.M., Airhart S.D., Strobel R.J., Keenan B.S. and Crawford J.D. (1981) Deletions of chromosome 15 as a cause of the Prader-Willi syndrome. N. Engl. J. Med. 304, 325–329 10.1056/NEJM198102053040604 7442771

[37] Isaacs J.S. and Zand D.J. (2007) Single-gene autosomal recessive disorders and Prader-Willi syndrome: an update for food and nutrition professionals. J. Am. Diet. Assoc. 107, 466–478 10.1016/j.jada.2006.12.006 17324666

[38] Butler M.G. (2009) Genomic imprinting disorders in humans: a mini-review. J. Assist. Reprod. Genet. 26, 477–486 10.1007/s10815-009-9353-3 19844787 PMC2788689

[39] Dykens E.M. and Roof E. (2008) Behavior in Prader-Willi syndrome: relationship to genetic subtypes and age. J. Child Psychol. Psychiatry 49, 1001–1008 10.1111/j.1469-7610.2008.01913.x 18665884

[40] Curfs L.M. and Fryns J.P. (1992) Prader-Willi syndrome: a review with special attention to the cognitive and behavioral profile. Birth Defects Orig. Artic. Ser. 28, 99–104 1340242

[41] Adams J.U. (2008) Imprinting and genetic disease: Angelman, Prader-Willi and Beckwith-Weidemann syndromes. Nat. Education 1, 129–130

[42] Ohta T., Gray T.A., Rogan P.K., Buiting K., Gabriel J.M., Saitoh S. et al. (1999) Imprinting-mutation mechanisms in Prader-Willi syndrome. Am. Human Genet. 64, 397–413 10.1086/302233 PMC1377750 9973278

[43] Wattendorf D.J. and Muenke M. (2005) Prader Willi Syndrome. Am. Fam. Physician 72, 827–830 16156341

[44] Bar C., Diene G., Molinas C., Bieth E., Casper C. and Tauber M. (2017) Early diagnosis and care is achieved but should be improved in infants with Prader-Willi syndrome. Orphanet J. Rare Dis. 12, 118, 10.1186/s13023-017-0673-6 28659150 PMC5490212

[45] Gross N., Rabinowitz R., Gross-Tsur V., Hirsch H.J. and Eldar-Geva T. (2015) Prader-Willi syndrome can be diagnosed prenatally. Am. J. Med. Gen. 167, 80–85 10.1002/ajmg.a.36812 25338954

[46] Bigi N., Faure J.-M., Coubes C., Puechberty J., Lefort G., Sarda P. et al. (2008) Prader-Willi syndrome: is there a recognizable fetal phenotype? Prenat. Diagn. 28, 796–799 10.1002/pd.1973 18661490

[47] Wijesuriya T.M., De Ceuninck L., Masschaele D., Sanderson M.R., Carias K.V., Tavernier J. et al. (2017) The Prader-Willi syndrome proteins MAGEL2 and necdin regulate leptin receptor cell surface abundance through ubiquitination pathways. Hum. Mol. Genet. 26, 4215–4230 10.1093/hmg/ddx311 28973533 PMC5886282

[48] Tacer K.F. and Potts P.R. (2017) Cellular and disease functions of the Prader-Willi Syndrome gene MAGEL2. Biochem. J. 474, 2177–2190 10.1042/BCJ20160616 28626083 PMC5594744

[49] Lee S., Walker C.L., Karten B., Kuny S.L., Tennese A.A., O'Neill M.A. et al. (2005) Essential role for the Prader-Willi syndrome protein necdin in axonal outgrowth. Hum. Mol. Genet. 14, 627–637 10.1093/hmg/ddi059 15649943

[50] Butler M., Bittel D., Kibiryeva N. and Garg U. (2006) C-reactive protein levels in subjects with Prader-Willi syndrome and obesity. Genet. Med. 8, 243–248 10.1097/01.gim.0000204469.30913.67 16617245 PMC5459599

[51] Chen W., Xu D., Ma C., Zhang C., Li J., Zhang W. et al. (2019) The molecular structure and imprinting status of the IPW (imprinted gene in the Prader-Willi syndrome region) gene in cattle. Anim. Genet. 50, 417–422 10.1111/age.12815 31268171

[52] Chen Z., Ju H., Yu S., Zhao T., Jing X., Li P. et al. (2018) Prader-Willi region non-protein coding RNA 1 suppressed gastric cancer growth as a competing endogenous RNA of miR-425-5p. Clin. Sci. (Lond.) 132, 1003–1019 10.1042/CS20171588 29535266

[53] Bieth E., Eddiry S., Gaston V., Lorenzini F., Buffet A., Conte Auriol F. et al. (2015) Highly restricted deletion of the SNORD116 region is implicated in Prader-Willi Syndrome. Eur. J. Hum. Genet. 23, 252–255 10.1038/ejhg.2014.103 24916642 PMC4297892

[54] Bischof J.M., Stewart C.L. and Wevrick R. (2007) Inactivation of the mouse Magel2 gene results in growth abnormalities similar to Prader-Willi syndrome. Hum. Mol. Genet. 16, 2713–2719 10.1093/hmg/ddm225 17728320

[55] Fountain M.D., Tao H., Chen C.A., Yin J. and Schaaf C.P. (2017) Magel2 knockout mice manifest altered social phenotypes and a deficit in preference for social novelty. Genes Brain Behav. 16, 592–600 10.1111/gbb.12378 28296079 PMC5495607

[56] https://www.fpwr.org/blog/new-insights-and-unexpected-connections-for-the-pws-gene-magel2

[57] Chen H., Victor A.K., Klein J., Tacer K.F., Tai D.J., de Esch C. et al. (2020) Loss of MAGEL2 in Prader-Willi syndrome leads to decreased secretory granule and neuropeptide production. JCI Insight 5, e138576 10.1172/jci.insight.138576 PMC7526459 32879135

[58] Oncul M., Dilsiz P., Ates Oz E., Ates T., Aklan I., Celik E. et al. (2018) Impaired melanocortin pathway function in Prader-Willi syndrome gene-Magel2 deficient mice. Hum. Mol. Genet. 27, 3129–3136 10.1093/hmg/ddy216 29878108

[59] Colmers W.F. and Wevrick R. (2013) Leptin signaling defects in a mouse model of Prader-Willi syndrome. Rare Dis. 1, e24421 10.4161/rdis.24421 25002992 PMC3927482

[60] MacDonald H.R. and Wevrick R. (1997) The Necdin Gene is deleted in Prader-Willi Syndrome and is Imprinted in Human and Mouse. Hum. Molar. Genet. 6, 1873–1878 10.1093/hmg/6.11.1873 9302265

[61] Miller N.L.G., Wevrick R. and Mellon P.L. (2009) Necdin, a Prader-Willi syndrome candidate gene, regulates gonadotropin-releasing hormone neurons during development. Hum. Mol. Genet. 18, 248–260 10.1093/hmg/ddn344 18930956 PMC2638776

[62] Polvora-Brandao D., Joaquim M., Godinho I., Aprile D., Alvaro A.R., Onofre I. et al. (2018) Loss of hierarchical imprinting regulation at the Prader-Willi/Angelman syndrome locus in human iPSCs. Hum. Mol. Genet. 27, 3999–4011 10.1093/hmg/ddy274 30102380 PMC6240739

[63] Valadares L.P., Meireles C.G., De Toledo I.P., Santarem de Oliveira R., Gonçalves de Castro L.C., Abreu A.P. et al. (2019) MKRN3 mutations in central precocious puberty: a systematic review and Meta-Analysis. J. Endocrine Soc. 3, 979–995 10.1210/js.2019-00041 31041429 PMC6483926

[64] Markson G., Kiel C., Hyde R., Brown S., Charalabous P., Bremm A. et al. (2009) Analysis of the human E2 ubiquitin conjugating enzyme protein interaction network. Genome Res. 19, 1905–1911 10.1101/gr.093963.109 19549727 PMC2765280

[65] Abreu A.P., Macedo D.B., Brito V.N., Kaiser U.B. and Latronico A.C. (2015) A new pathway in the control of the initiation of puberty: the MKRN3 gene. J. Mol. Endocrinol. 54, R131–R139 10.1530/JME-14-0315 25957321 PMC4573396

[66] Neumann L.C., Markaki Y., Mladenov E., Hoffmann D., Buiting K. and Horsthemke B. (2012) The imprinted NPAP1/C15orf2 gene in the Prader-Willi syndrome region encodes a nuclear pore complex associated protein. Hum. Mol. Genet. 21, 4038–4048 10.1093/hmg/dds228 22694955

[67] Neumann L.C., Feiner N., Meyer A., Buiting K. and Horsthemke B. (2004) The imprinted NPAP1 gene in the Prader-Willi syndrome region belongs to a POM121-related family of retrogenes. Genome Biol. Evol. 6, 344–351 10.1093/gbe/evu019 PMC3942032 24482533

[68] Cao Y., AlHumaidi S.S., Faqeih E.A., Pitel B.A., Lundquist P. and Aypar U. (2017) A novel deletion of SNURF/SNRPN exon 1 in a patient with Prader-Willi-like phenotype. Eur. J. Med. Genet. 60, 416–420 10.1016/j.ejmg.2017.05.003 28554868

[69] Runte M., Hüttenhofer A., Groß S., Kiefmann M., Horsthemke B. and Buiting K. (2001) The IC-SNURF-SNRPN transcript serves as a host for multiple small nucleolar RNA species and as an antisense RNA for UBE3A. Hum. Molec. Genet. 10, 2687–2700 10.1093/hmg/10.23.2687 11726556

[70] Mendiola A.J.P. and LaSalle J.M. (2021) Epigenetics in Prader-Willi syndrome. Front. Genet. 12, 1–13, Article-624581 10.3389/fgene.2021.624581 PMC7917289 33659026

[71] Mian-Ling Z., Yun-Qi C. and Chao-Chun Z. (2020) Prader-Willi syndrome: molecular mechanism and epigenetic therapy. Curr. Gene. Therapy 20, 36–43 10.2174/1566523220666200424085336 32329685

[72] Cruvinel E. Budinetz T., Germain N., Chamberlain S., Lalande M., Martins-Taylor K. (2014) Reactivation of maternal SNORD116 cluster via SETDB1 knockdown in Prader-Willi syndrome iPSCs. Hum Mol Genet. 23, 4674–4685 10.1093/hmg/ddu187 24760766 PMC4481691

[73] Langouët M., Glatt-Deele H.R., Chung M.S., Dupont-Thibert C.M., Mathieux E., Banda E.C. et al. (2018) Zinc finger protein 274 regulates imprinted expression of transcripts in Prader-Willi syndrome neurons. Hum. Mol. Genet. 27, 505–515 10.1093/hmg/ddx420 29228278

[74] Langouët M., Gorka D., Orniacki C., Dupont-Thibert C.M., Chung M.S., Glatt-Deele H.R. et al. (2020) Specific ZNF274 binding interference at SNORD116 activates the maternal transcripts in Prader-Willi syndrome neurons. Hum. Mol. Genet. 29, 3285–3295 10.1093/hmg/ddaa210 32977341 PMC7689300

[75] Turpeinen H., Ortutay Z. and Pesu M. (2013) Genetics of the first seven proprotein convertase enzymes in health and disease. Curr. Genomics 14, 453–467 10.2174/1389202911314050010 24396277 PMC3867721

[76] Ramos-Molina B., Martin M.G. and Lindberg I. (2016) PCSK1 variants and human obesity. Prog. Mol. Biol. Transl. Sci. 140, 47–74 10.1016/bs.pmbts.2015.12.001 27288825 PMC6082390

[77] Lin J., Wu H.-T., Qin X.-Y. and Lan R. (2017) Dissecting carboxypeptidase E: properties, functions and pathophysiological roles in disease. Endocr. Connect. 6, R18–R38 10.1530/EC-17-0020 28348001 PMC5434747

[78] Jackson R.S., Creemers J.W.M., Farooqi S., Raffin-Sanson M.-L., Varro A., Dockray G.J. et al. (2003) Complex endocrinopathy of human proprotein convertase 1 deficiency. J. Clin. Invest. 112, 1550–1560 10.1172/JCI200318784 14617756 PMC259128

[79] Scamuffa N., Calvo M., Chrétien M., Seidah N.G. and Khatib A.-M. (2006) Proprotein convertases: lessons from knockouts. FASEB J. 20, 1954–1963 10.1096/fj.05-5491rev 17012247

[80] Burnett L.C., LeDuc C.A., Sulsona C.R., Paull D., Rausch R., Eddiry S. et al. (2017) Deficiency in prohormone convertase PC1 impairs prohormone processing in Prader-Willi syndrome. J. Clin. Invest. 127, 293–305 10.1172/JCI88648 27941249 PMC5199710

[81] Burman P., Ritzén E.M. and Lindgren A.C. (2001) Endocrine dysfunction in Prader-Willi syndrome: a review with special reference to GH. Endocr. Rev. 22, 787–799 10.1210/edrv.22.6.0447 11739333

[82] Chrétien M. and Mbikay M. (2016) 60 YEARS OF POMC: from the prohormone theory to pro-opiomelanocortin and to proprotein convertases (PCSK1 to PCSK9). J. Mol. Endocrinol. 56, T49–T62 10.1530/JME-15-0261 26762158

[83] Day R., Lazure C., Basak A., Boudreault A., Limperis P., Dong W. et al. (1998) Prodynorphin processing by proprotein convertase 2. Cleavage at single basic residues and enhanced processing in the presence of carboxypeptidase activity. J. Biol. Chem. 273, 829–836 10.1074/jbc.273.2.829 9422738

[84] Polex-Wolf J., Yeo G.S.H. and O'Rahilly S. (2017) Impaired prohormone processing: a grand unified theory for features of Prader-Willi syndrome? J. Clin. Invest. 127, 98–99 10.1172/JCI91307 27941250 PMC5199707

[85] Wang L., Sui L., Panigrahi S.K., Meece K., Xin Y., Kim J. et al. (2017) PC1/3 deficiency impacts pro-opiomelanocortin processing in human embryonic stem cell-derived hypothalamic neurons. Stem Cell Rep. 8, 264–277 10.1016/j.stemcr.2016.12.021 28132887 PMC5312251

